# Ferrierite and Its Delaminated Forms Modified with Copper as Effective Catalysts for NH_3_-SCO Process

**DOI:** 10.3390/ma13214885

**Published:** 2020-10-30

**Authors:** Aneta Święs, Małgorzata Rutkowska, Andrzej Kowalczyk, Urbano Díaz, Antonio E. Palomares, Lucjan Chmielarz

**Affiliations:** 1 Faculty of Chemistry, Jagiellonian University in Kraków, Gronostajowa 2, 30-387 Kraków, Poland; aneta.swies@doctoral.uj.edu.pl (A.Ś.); ma.rutkows@gmail.com (M.R.); kowalczy@chemia.uj.edu.pl (A.K.); 2 Instituto de Tecnología Química, Universitat Politècnica de València–Consejo Superior de Investigaciones Científicas, Avd. de los Naranjos s/n, 46022 Valencia, Spain; udiaz@itq.upv.es (U.D.); apalomar@iqn.upv.es (A.E.P.)

**Keywords:** NH_3_-SCO, ferrierite, ITQ-6, aluminum, titanium, copper

## Abstract

Ferrierites and their delaminated forms (ITQ-6), containing aluminum or titanium in the zeolite framework, were synthetized and modified with copper by an ion-exchange method. The obtained samples were characterized with respect to their chemical composition (ICP-OES), structure (XRD, UV-Vis DRS), textural parameters (N_2_-sorption), surface acidity (NH_3_-TPD), form and reducibility of deposited copper species (UV-Vis DRS and H_2_-TPR). Ferrierites and delaminated ITQ-6 zeolites modified with copper were studied as catalysts for the selective catalytic oxidation of ammonia to dinitrogen (NH_3_-SCO). It was shown that aggregated copper oxide species, which were preferentially formed on Ti-zeolites, were catalytically active in direct low-temperature ammonia oxidation to NO, while copper introduced into Al-zeolites was present mainly in the form of monomeric copper cations catalytically active in selective reduction of NO by ammonia to dinitrogen. It was postulated that ammonia oxidation in the presence of the studied catalysts proceeds according to the internal-selective catalytic reduction mechanism (i-SCR) and therefore the suitable ratio between aggregated copper oxide species and monomeric copper cations is necessary to obtain active and selective catalysts for the NH_3_-SCO process. Cu/Al-ITQ-6 presented the best catalytic properties possibly due to the most optimal ratio of these copper species.

## 1. Introduction

Ammonia belongs to dangerous air pollutants. The global ammonia emission to atmospheres has been estimated to be on the level of 44 million tons per year (excluding oceanic sources) [1]. Agriculture is the main anthropogenic source of ammonia emission and was estimated to contribute about 75% of the global ammonia emission (excluding oceanic sources) [1]. The other 25% is related mainly to biomass conversion, including biofuels production (about 13%), industrial processes (about 0.5%) and fossil fuels combustion (about 0.2%) [1]. Other ammonia emission sources are poorly documented [1]. The contribution of ammonia emissions from industrial processes and fossil fuels combustion seems to be negligible in the global emission of ammonia, however it is prognosed that the role of these sources will increase in the future, especially in the case of fuel combustion in the transportation sector [2]. It is assigned to the common use of three-way catalysts for purification of flue gases in cars with spark ignition engines, which increases ammonia emission [3]. In the case of cars with diesel engines, the diesel exhaust fluid (DEF) technology, called in Europe AdBlue, has been implemented. This technology is based on the reduction of nitrogen oxides present in flue gases by ammonia. In this case ammonia is produced on-board by urea hydrolysis [4]. Thus, increased ammonia emission by diesel cars is also expected in the future. Moreover, ammonia is used as an NO_x_ reduction agent in flue gases emitted by stationary sources, such as electric power stations [5,6], thus in this case there is also a risk of ammonia leaks.

One of the most promising technologies for the elimination of ammonia from flue gases is based on selective catalytic oxidation of ammonia to dinitrogen, NH_3_-SCO [7,8]. In this case the role of the oxidising agent plays oxygen present in flue gases and therefore there is no need for additional reactant introduction to the gas stream. The main problem is related to development of the effective catalysts operating in the temperature of flue gases with high selectivity to dinitrogen. The catalytic systems studied in the process of selective ammonia oxidation [8] are divided into three main groups: (1) noble metals, which are active in relatively low temperatures but are low-selective to dinitrogen [9,10,11]; (2) transition metal oxides, typically active at higher temperatures but more selective to dinitrogen than noble metals [12,13,14]; and (3) zeolites modified with transition metals, which are catalytically active at temperatures higher than noble metals but are significantly more selective to dinitrogen [15,16,17].

The presented studies are focused on the determination of the catalytic performance of ferrierites containing aluminum or titanium introduced into the zeolite framework as well as their delaminated forms (ITQ-6) doped with copper in the process of selective catalytic oxidation of ammonia to dinitrogen and water vapour. Ferrierite is microporous three-dimensional zeolite, while ITQ-6 is a delaminated zeolite composed of microporous ferrierite layers randomly oriented with the formation of the structure called “house of card”. In this case, micropores are located within speared ferrierite layers, while the spaces between randomly oriented layers are mesopores. The various porous structures, microporous in the case of ferrierite and bimodal microporous and mesoporous in the case of ITQ-6, should not only influence the internal diffusion rate of reactants inside pores but also forms of deposited copper species, which, depending on aggregation, may play a different role in the studied process [8]. Aggregated copper oxide species were reported to be catalytically active in direct ammonia oxidation to NO [18], thus the presence of such species may activate catalysts for the low-temperature ammonia oxidation but to the undesired reaction product. On the other side, highly dispersed copper species, mainly monomeric copper cations, are known to be catalytically active in the process of selective catalytic reduction of NO with ammonia to dinitrogen, NH_3_-SCR, [17]. Thus, NO formed by direct ammonia oxidation over aggregated copper oxide species in the next step could be reduced by residual ammonia to dinitrogen in the presence of highly dispersed copper species. Such mechanism of ammonia oxidation, called internal selective catalytic reduction, i-SCR, was proved for various catalytic systems [8]. It should be noted that for the formulation of the active, selective and stable catalysts for ammonia oxidation the suitable contribution of dispersed and aggregated copper species in the catalyst is necessary.

Thus, in the present studies the type of porous structure of the zeolitic catalysts as well as the presence of titanium and aluminum in the zeolite framework on the nature of deposited copper species and therefore catalytic activity in selective ammonia oxidation process was analysed. 

## 2. Materials and Methods 

### 2.1. Catalysts Preparation

Synthesis of ferrierite and ITQ-6 zeolites, containing aluminum or titanium in the zeolite framework, was done according to the procedures presented earlier [19,20]. The brief descriptions of the all syntheses steps are presented below. 

#### 2.1.1. Synthesis of Zeolite Precursors

Al-PREFER and Ti-PREFER, the zeolite precursors with aluminum or titanium incorporated in the zeolite framework, were synthesized using fumed silica Aerosil 200 (Evonik Industries AG, Essen, Germany) as silicon source, hydroxy(oxo)alumane Catapal B (Sasol, Johannesburg, South Africa), as aluminum source and titanium (IV) ethoxide (98%, Alfa Aesar, Haverhill, MA, USA) as titanium source. 4-amino-2,2,6,6-tetramethylpiperidine (R, Fluka, München, Germany) was used as structure directing agent. Moreover, NH_4_F (98%, Sigma-Aldrich, St. Louis, MO, USA), HF (49.8%, Sigma-Aldrich, St. Louis, MO, USA) and distilled water were used for the zeolite synthesis. Molar ratio of components was 1 SiO_2_: 0.01 Al_2_O_3_ (or 0.02 TiO_2_): 1 R: 1.5 NH_4_F: 0.5 HF: 10 H_2_O, resulting in Si/Al or Si/Ti molar ratio of 50. The obtained slurries were agitated in an autoclave at 135 °C for 3 days in the case of Al-PREFER and for 11 days in the case Ti-PREFER. The final steps were filtration, washing with distilled water and drying at 60 °C for 24 h.

#### 2.1.2. Synthesis of Al-FER and Ti-FER

Calcination of Al-PREFER and Ti-PREFER at 600 °C for 6 h resulted in layers condensation leading to three dimensional (3D) ferrierites, called Al-FER and Ti-FER, respectively.

#### 2.1.3. Swelling of PREFER and ITQ-6 Synthesis

The second part of Al-PREFER and Ti-PREFER was swollen in a solution of 25 g hexadecyltrimethylammonium bromide (CTMABr, 50% exchanged Br/OH, ≥99%, Acros Organics, Geel, Belgium) and 12 g tetrapropylammonium bromide (TPABr, 30% exchanged Br/OH, 98% Sigma-Aldrich, St. Louis, MO, USA) diluted in 113 g of distilled water. 5 g of Al-PREFER or Ti-PREFER was immersed in alkylammonium surfactants solution and stirred under reflux for 16 h at 95 and 80 °C for Al-PREFER and Ti-PREFER, respectively. Then, the expanded Al-PREFER and Ti-PREFER samples were delaminated by sonication of the slurries in an ultrasound bath (50 W, 40 kHz) for 1 h. In the next step, the slurries were acidified to pH = 3.0 using concentrated HCl (high purity grade, Honeywell, Charlotte, NC, USA), washed with water and separated by centrifuging (12,000 rpm, 20 min). The resulting solid was calcined at 600 °C for 6 h in air atmosphere, resulting in Al-ITQ-6 and Ti-ITQ-6.

#### 2.1.4. Modification of Zeolites with Copper

Copper was introduced to ferrierites, Al-FER and Ti-FER, as well as their delaminated forms, Al-ITQ-6 and Ti-ITQ-6, by an ion-exchange method. The zeolitic samples were introduced to 0.06 M aqueous solution of Cu(CH_3_COO)_2_·H_2_O (10 g of solid sample per 1000 g of solution) and the obtained slurries were stirred at 80 °C for 6 h. Then, the samples were filtrated, washed with distilled water and dried at 60 °C for 24 h. Finally, copper modified zeolites were calcined at 550 °C for 8 h, resulting in the samples denoted as: Cu/Al-FER, Cu/Ti-FER, Cu/Al-ITQ-6 and Cu/Ti-ITQ-6.

### 2.2. Catalysts Characterization

Specific surface area and pore volumes of the zeolitic samples were analyzed by dinitrogen sorption at −196 °C using 3Flex (Micrometrics, Norcross, GA, USA) in an automated gas adsorption system. Before the measurements, the samples were outgassing under vacuum at 350 °C for 24 h. Profiles of micropore and mesopore size distribution were determined using H-K (Horvath-Kawazoe) and BJH (Barrett, Joyner and Halenda) models, respectively. The total pore volume calculation was based on the total amount of adsorbed dinitrogen at relative pressure p/p_0_ = 0.98. The structure of the studied samples was characterized by an X-ray diffraction method using a Bruker D2 Phaser diffractometer (Bruker, Billerica, MA, USA). The following measurement settings were applied: Cu-Kα radiation with λ = 0.154056 nm, the 2 theta range of 5–40° with step scans of 0.02° and a counting time of 1 s per step.

An Inductively Coupled Plasma-Optical Emission Spectrometry (ICP-OES) method was used for the analysis of the chemical composition of the samples. Acidic solutions consisting of hydrofluoric acid (high purity grade, Honeywell, Charlotte, NC, USA), hydrochloric acid (high purity grade, Honeywell, Charlotte, NC, USA) and nitric acid (high purity grade, Honeywell, Charlotte, NC, USA) were used for the solid samples dissolution. This process was conducted with the assistance of microwave radiation using Ethos Easy system (Milestone, Sorisole, Italy). The obtained solutions were analyzed using an iCAP 7400 instrument (Thermo Scientific, Waltham, MA, USA).

The form of titanium and copper species present in the zeolitic samples were analyzed by UV-vis-DR spectroscopy (Evolution 600 Thermo Scientific, Waltham, MA, USA) in the range of 200–900 nm with a resolution of 2 nm. Surface concentration of acid sites in the samples as well as their relative strength were analyzed by temperature-programmed desorption of ammonia (NH_3_-TPD). The sample (50 mg) was placed in a fixed-bed quartz microreactor and, prior to the NH_3_-TPD run, outgassed in a flow of pure helium (20 mL/min) at 600 °C until water was completely evacuated for the sample (monitored by a quadrupole mass spectrometer–QMS, PREVAC, Rogów, Poland). In the next step, after cooling the sample to 70 °C, a flow of helium was exchanged for mixture of 1 vol.% ammonia diluted in helium (20 mL/min). After saturation of the sample with ammonia, the reactor was purged with pure helium to remove physiosorbed forms of ammonia (both steps monitored by QMS). Finally, the NH_3_-TPD measurement was carried out in the temperature range of 70–600 °C with heating rate of 10 °C/min in a flow of pure helium (20 mL/min). Desorbing ammonia molecules were monitored using a quadrupole mass spectrometer (PREVAC, Rogów, Poland) connected directly to the reactor outlet. The reducibility of the samples modified with copper was studied by temperature–programmed reduction with hydrogen used as a reducing agent (H_2_-TPR). The sample (50 mg), placed in a fixed-bed quartz microreactor, was outgassed in a flow of pure argon (10 ml/min) at 500 °C for 30 min. The H_2_-TPR run was carried out in a flow of 5.0 vol.% H_2_ diluted in argon (10 mL/min) from 100 to 900 °C with the linear heating rate of 10 °C/min. A thermal conductivity detector (TCD, VICI, Houston, TX, USA) was used for the analysis of hydrogen concentration in outlet gases.

### 2.3. Catalytic Studies

Zeolitic catalysts modified with copper were tested as catalysts for the selective catalytic oxidation of ammonia (NH_3_-SCO) in a flow microreactor system. The catalysts sample of 100 mg, placed in a quartz microreactor, was outgassed in a flow of helium (20 mL/min) at 550 °C for 1 h. The NH_3_-SCO catalytic test was carried in the temperature range of 200–550 °C with a heating rate of 10 °C/min using gas mixture containing: 0.5 vol.% NH_3_ and 2.5 vol.% O_2_ diluted in helium. Concentrations of reactants were measured by a quadrupole mass spectrometer-QMS (PREVAC, Rogów, Poland) connected via a heated line to the reactor outlet.

## 3. Results and Discussion 

### 3.1. Characterization of Catalyst Precursors and Catalysts

The measured molar Si/Al and Si/Ti ratios, presented in Table 1, are 64 and 60 for Al-FER and Ti-FER, respectively. Thus, the real Si/Al and Si/Ti ratios are higher than the intended value of 50, indicating than not all aluminum and titanium was incorporated into the zeolite frameworks. In delaminated ITQ-6 zeolites, the Si/Al and Si/Ti ratios decreased, possibly due to treatment of the zeolite precursors in acidic media (pH = 3) in the final step of delamination process.

#### 3.1.1. X-ray Diffraction

**Figure 1:**
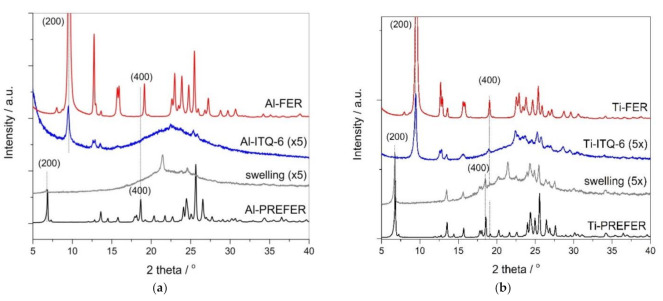

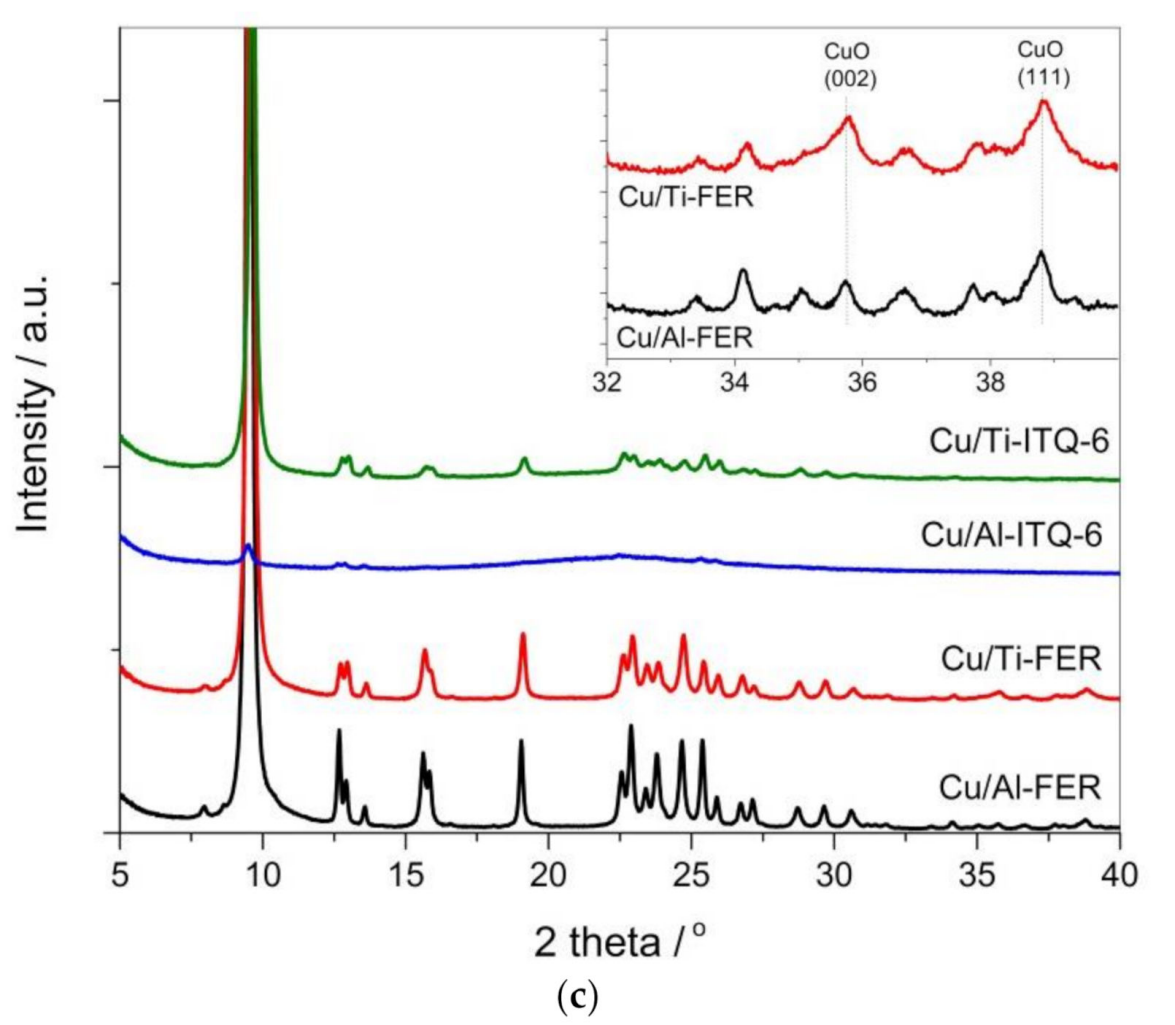
X-ray diffractograms recorded for the ferrierite-based samples (PREFER, swellen PREFER, delaminated ITQ-6 and FER) with aluminum (**a**) and titanium (**b**) incorporated into the zeolite framework as well as their modifications with copper (**c**).

The structure of zeolite catalysts on different stages of their synthesis was analyzed by the XRD method. In diffractogram of Al-PREFER precursor, presented in Figure 1a, the (200) diffraction peak at 2Θ = 6.8°, indicating the interlayer distance of 1.3 nm, was identified [19,20]. In the diffractogram of calcined Al-PREFER the (200) reflection at 2Θ = 9.6°, characteristic of condensated zeolite layers in ferrierite, appeared. Other peaks in diffractogram of Al-FER are typical of the ferrierite structure and prove the successful synthesis of this zeolite [21]. On the other side, the swelling of Al-PREFER resulted in reduction of the (200) reflection, indicating non-parallel orientation of the zeolite layers in the swollen sample. Moreover, the intensity of the other reflections was significantly reduced and some reflections even disappeared. It shows that Al-PREFER is not fully stable under strongly basic conditions of swelling. This hypothesis is supported by the presence of the broad diffraction peak spread at about 15–30° characteristic of amorphous silica [22]. Possibly, a part of silicon, leached from the zeolite layers under swelling conditions, was precipitated in a solution and re-deposited on the external zeolite surface in the form of amorphous silica. Sonication followed by calcination of such swollen Al-PREFER samples resulted in delaminated Al-ITQ-6 zeolite. The low-intensive reflection at 2Θ = 9.6°, characteristic of the ferrierite structure, indicates that a small fraction was not effectively separated by swelling process and condensated during calcination. However, comparison of the (200) reflection intestines in diffractograms of Al-FER and Al-ITQ-6 indicates that the contribution of the ferrierite phase in the later sample is very small and in this case the delaminated structure dominated. 

The (200) diffraction peak in Ti-PREFER diffractogram, similarly to Al-PREFER, is located at 2Θ about 6.8° (Figure 1b), indicating an interlayer distance of about 1.3 nm. Ti-PREFER swelling resulted in a decrease of intensity and a small shift of the (200) reflection to about 6.6°, indicating delamination of the layered zeolite structure with the small contribution of the parallel layers with slight increases in the interlayer distance. It should be noted that the reflection characteristics of intralayer ordering are significantly more intensive for swollen Ti-PREFER than for swollen Al-PREFER, indicating that the ferrierite layers containing titanium are more stable under basic conditions of swelling compared to the aluminum-containing ferrierite layers. However, a broad diffraction peak at about 15–30° also shows in this case that a part of silicon was dissolved from the ferrierite layers and formed amorphous silica [22]. Sonication and calcination of swollen Ti-PREFER resulted in delaminated Ti-ITQ-6 with the small contribution of the ferrierite phase, identified by the low-intensive (200) reflection at 2Θ of about 9.6°. Direct calcination of Ti-PREFER resulted in the formation of the ferrierite zeolite (Ti-FER), which was verified by the presence of the characteristic reflections in a diffractogram of this sample [21].

A diffractogram recorded for the zeolitic samples doped with copper are presented in Figure 1c. The reflections characteristic of zeolite structures were not changed after copper deposition. However, in the case of Al-FER and Ti-FER there were low-intensive reflections, indicating the presence of small CuO crystallites, which were identified (Figure 1c). Such crystallites, due to the microporous character of ferrierite, are located outside the porous system of the samples. In diffractograms of Cu-Al-ITQ-6 and Cu-Ti-ITQ-6, such reflections are not present.

#### 3.1.2. N_2_ Sorption

**Figure 2:**
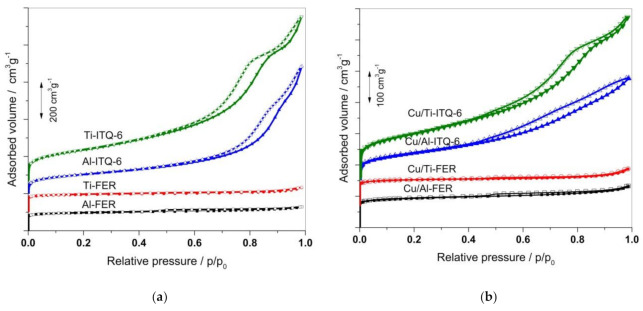
Dinitrogen isotherms recorded for Al- and Ti-containing zeolite samples (**a**) and their Cu-modified forms (**b**).

Dinitrogen adsorption–desorption isotherms of the zeolitic samples and their modifications with copper are shown in Figure 2. The isotherms measured for ferrierites, Al-FER and Ti-FER, and presented in Figure 2a, are classified as type I according to the IUPAC recommendations [23] and are typical of microporous materials. An adsorption step at a very low relative pressure is related to capillary condensation of dinitrogen inside micropores and proves the microporous character of the samples. The isotherms measured for Al-ITQ-6 and Ti-ITQ-6 are classified as type IV according to the IUPAC standards [23] and are typical of mesoporous materials (Figure 2a). An adsorption step observed in low range of the relative pressure indicates the presence of micropores in this series of the samples. Thus, the isotherms of Al-ITQ-6 and Ti-ITQ-6 show a bimodal type of porosity in these samples. Micropores are channels in the zeolite layers, while mesopores are spaces between non-parallel oriented zeolite layers. The hysteresis loops present in isotherms of Al-ITQ-6 and Ti-ITQ-6 are classified as H3 and are characteristic of non-rigid aggregates of plate-like particles [23]. The neckings in the hysteresis loops indicate partial plugging of mesopores [24]. Deposition of copper into the zeolite samples did not result in significant modifications of their isotherm profiles (Figure 2b).

**Figure 3:**
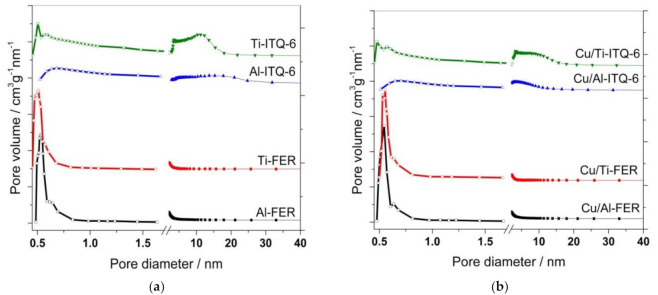
Pore size distributions determined for Al-, Ti-zeolites in micropore and mesopore range (**a**) and their Cu-modified forms (**b**).

Pore size distributions (PDS) of the zeolitic samples and their modifications with copper in micro and mesopore regions are presented in Figure 3. For the ferrierite samples, Al-FER and Ti-FER, the main maximum is at about 0.50–0.53 nm. The position of these maxima correlates with the diameter of 10 MR (10 member ring) channels in ferrierites [25]. Any peaks in PSD of Al-FER and Ti-FER were found in the mesoporore range, proving the microporous character of these samples. Deposition of copper into these samples did not result in significant modifications of the PSD profiles (Figure 3). In the case of Al-ITQ-6 and Ti-ITQ-6, the maxima of PSD in the micropore range were significantly reduced in the comparison to the ferrierite samples, which is assigned the limitation of the microporous structure only to zeolite layers for Al-ITQ-6 and Ti-ITQ-6, in contrast to the microporous three-dimensional structures of Al-FER and Ti-FER. The long tails from the side of larger micropores, present in the PSD profiles of Al-ITQ-6 and Ti-ITQ-6, show significant heterogeneity in micropore size distribution caused by partial destruction of the ferrierite layers under swelling conditions. In the mesopore range the broad maxima, typical of the delaminated structure, are present in PSD profiles of delaminated zeolites (Figure 3). In the case of Al-ITQ-6, the maximum is located at about 5–6 nm with the long tail from the larger mesopore side. The PSD profile of Ti-ITQ-6 is more complex and consists of sharp peak at about 3.6 nm and a broad maximum centred at about 10–11 nm. As it was shown by XRD studies (Figure 1), under swelling conditions, part of the silica was removed from the ferrierite layers and re-deposited on the zeolite external surface in the form of amorphous silica. Thus, the contribution of mesopores in such amorphous silica should be taken into account. Deposition of copper into Ti-ITQ-6 decreased the intensity of broad maximum of PSD, indicating preferential location of copper species inside larger pores.

Textural parameters of the samples are compared in Table 1. In the case of Al-FER and Ti-FER, the specific surface area (S_BET_) is about 370 m^2^/g, while the micropore volume (V_micro_) is about 0.13–0.14 cm^3^/g. Mesopore volume measured for these samples is possibly related to the inter-crystalline and inter-grain spaces. Delamination of the ferrierite samples, resulting in Al-ITQ-6 and Ti-ITQ-6, opened interlayer spaces, which was indicated by a decrease in micropore volumes to about 0.02 cm^3^/g and an increase in the mesopore volume by more than one order of magnitude. The BET surface area is significantly larger for Ti-ITQ-6 than for Al-ITQ-6. This is possibly related to a more significant contribution of mesoporous silica in the Ti-ITQ-6 sample. Deposition of copper into Al-FER and Ti-FER resulted in a decrease of the BET surface area by about 5 and 10%, respectively. Moreover, a small decrease in the micropore volumes was observed. Introduction of copper into Al-ITQ-6 and Ti-ITQ-6 decreased their BET surface area by about 24 and 15%, respectively. The analysis of micropore and mesopore volumes for these samples before and after copper deposition shows that metal species were deposited mainly in mesopores, possibly also in aggregated forms, which is indicated by significantly decreased mesopore volume for the copper doped samples (Table 1).

#### 3.1.3. UV-Vis DR Spectroscopy

**Figure 4:**
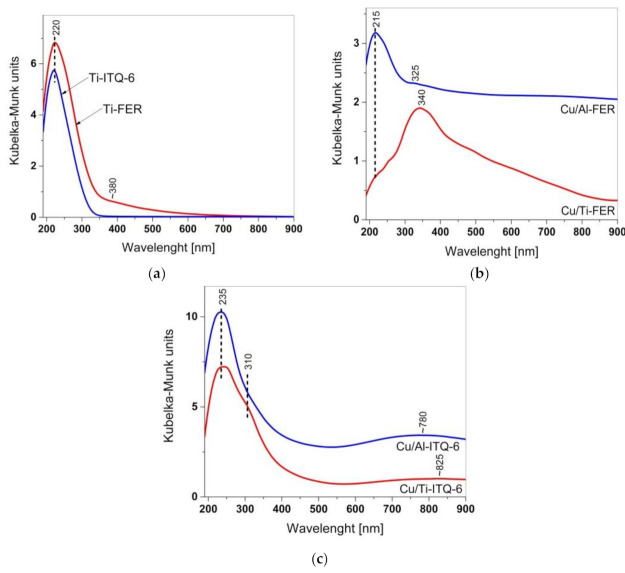
UV-vis DR spectra of Ti-zeolites (**a**), ferrierites modified with copper (**b**) and ITQ-6 zeolites modified with copper (**c**).

The form and aggregation of titanium and copper in the zeolitic samples were analysed by UV-Vis DR spectroscopy (Figure 4). In the spectra of Ti-FER and Ti-ITQ-6, shown in Figure 4a, the maxima at about 220 nm are attributed to monomeric tetrahedrally coordinated titanium cations in the zeolite framework [21,26]. Small shoulder above 350 nm in a spectrum of Ti-ITQ-6 is related to extra framework titanium species of various aggregations, from small polymerized hexacoordinated Ti-species containing Ti-O-Ti bridges to small aggregates of TiO_2_ [21,26]. Thus, in Ti-FER titanium is present in the form of tetrahedrally coordinated Ti^4+^ cations incorporated into the zeolite framework. This same form of titanium dominates in Ti-ITQ-6, however in this case there is also a small contribution of titanium in the form of extra framework species.

The UV-vis DR spectra of the zeolite samples modified with copper are shown in Figure 4b,c. In the case of Cu/Ti-FER and Cu/Ti-ITQ-6, there are differential spectra, obtained by the subtraction of the Ti-FER spectrum from the original spectrum of Cu/Ti-FER, and analogously by substation of Ti-ITQ-6 spectrum from the spectrum of Cu/Ti-ITQ-6. Such differential spectra are characteristic of the copper species present in the samples. In the case of Cu/Al-FER and Cu/Al-ITQ-6, copper is the only element sensitive in UV-Vis irradiation. The spectra recorded for Cu/Al-FER and Cu/Ti-FER differ significantly (Figure 4b). In the case of Cu/Al-FER the main absorption peak is located at about 215 nm and is related to the presence of monomeric copper ions interacting with the oxygen of the zeolite framework (O^2−^→Cu^2+^). Thus, this band indicates the presence of monomeric copper cations possibly located in the ion-exchange sites of zeolite [19]. A small shoulder at about 325 nm indicates small oligomeric copper oxide species, while a shoulder at higher wavelengths is characteristic of small CuO aggregates [19]. This is consistent with the result of the XRD analysis, which shows the presence of the reflections characteristic of CuO in a diffractogram of the Cu/Al-FER sample (Figure 1c). The presence of monomeric copper cations in Cu/Ti-FER is assigned to small shoulder at about 215 nm. The main absorption peak, located at about 340 nm, indicates a significant contribution of oligomeric copper oxide species, which possibly dominate in this sample. The shoulder above 400 nm is assigned to CuO crystallites [27]. The presence of such crystallites in the Cu/Ti-FER sample was proved by XRD studies (Figure 1c). In the spectra of Cu/Al-ITQ-6 and Cu/Ti-ITQ-6 (Figure 4c), the bands at about 235 nm, characteristic of monomeric coper cations, dominate. The presence of such highly dispersed copper cations is proved by the broad bands above 550 nm, indicating d-d transition in surface Cu^2+^ cations in pseudo-octahedral coordination interacting with water molecules [28,29]. The shoulder at about 310 nm, more intensive for Cu/Ti-ITQ-6, is assigned to oligomeric copper oxides species [28,29].

Such significant differences in various copper species deposited into the zeolite samples are possibly determined mainly by their porous structure and interaction with the zeolite framework. Al-FER and Ti-FER are characterized by a microporous structure consisting of 10 MR channels with the diameter of about 0.52 nm [25]. The size of the octahedral aqua copper complex, [Cu(H_2_O)_6_]^2+^, is very similar to the diameter of 10 MR [30], and therefore internal diffusion of such aqua complexes of copper in 10 MR is limited. Thus, in these zeolite samples, copper species are deposited mainly on the external surface of the zeolite crystallites and during calcination form more aggregated copper species. This effect was less significant for Cu/Al-FER because of lower copper loading in this sample. On the other side, Al-ITQ-6 and Ti-ITQ-6 contain both micropores and mesopores, and there are no diffusional restrictions for the [Cu(H_2_O)_6_]^2+^ complex in mesopores. Thus, in this case, interlayer space is available for such complexes, which results in the deposition of copper in the form of highly dispersed species.

#### 3.1.4. H_2_-TPR

**Figure 5:**
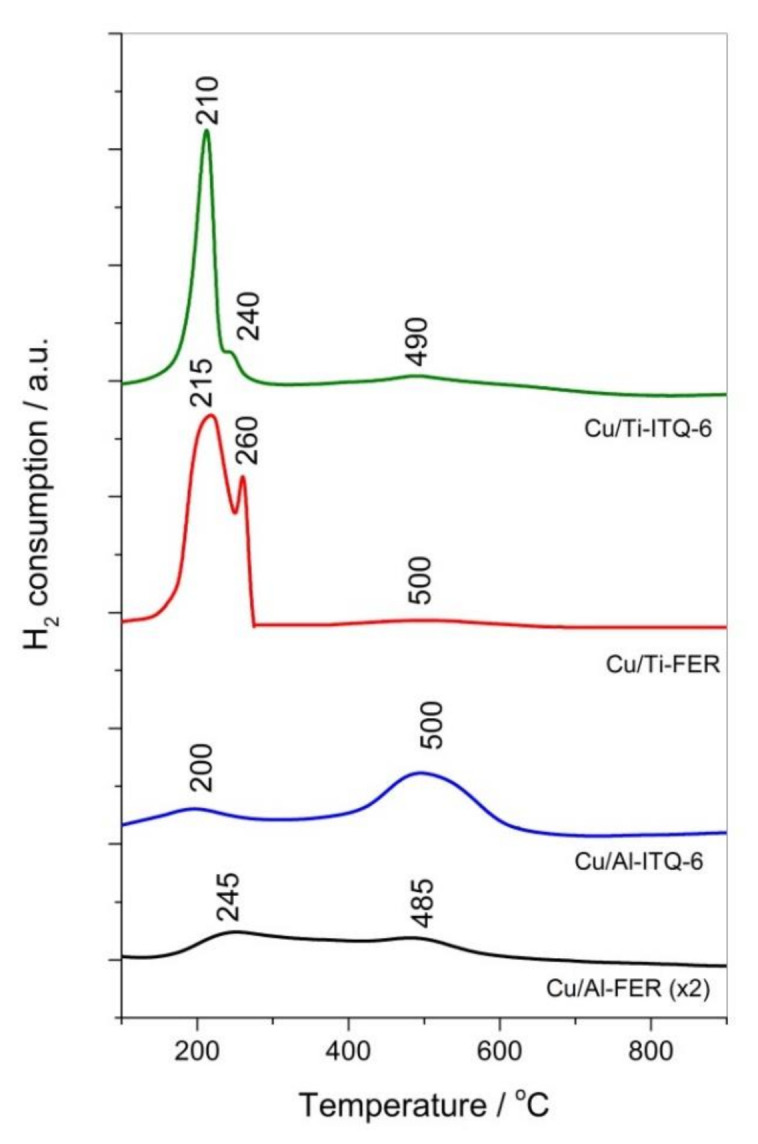
H_2_-TPR profiles of ferrierite and ITQ-6 zeolites modified with copper.

Reducibility of copper species introduced into the zeolite samples was analyzed by a method of temperature-programmed reduction using hydrogen as a reducing agent (H_2_-TPR). The mechanisms of the copper species reduction strongly depend on their form and aggregation. At temperatures below 300 °C, the reduction of Cu^2+^ to Cu^0^ in aggregated copper oxide species and CuO crystals takes place. Reduction of monomeric Cu^2+^ cations, e.g., in ion-exchange positions, includes two stages. In the first step, below 300 °C, the reduction of Cu^2+^ to Cu^+^ occurs. While in the second step, Cu^+^ to Cu^0^ is reduced at higher temperatures, typically above 400 °C [19,31]. Thus, the results of H_2_-TPR are important for an analysis of the form and aggregation of copper species present in catalysts. Reduction profiles of the copper modified ferrierites, Cu/Al-FER and Cu/Ti-FER, consist of low temperature peaks, which represent both reduction of Cu^2+^ to Cu^0^ in aggregated copper species and reduction of monomeric Cu^2+^ cations to Cu^+^ (Figure 5). The high temperature broad peaks, assigned to the reduction of monomeric Cu^+^ cations to Cu^0^, are located at about 485 °C for Cu/Al-FER and 500 °C for Cu/Ti-FER. The intensity of the Cu/Al-FER reduction profile is lower due to the lower copper loading in this sample. Moreover, low-temperature maximum is much more intensive in the case of Cu/Ti-FER and consists of at least two peaks, possibly indicating a reduction of the copper species of various aggregations. Integration and comparison of the surface areas under low and high temperature maxima leads to an evaluation of copper contribution in the form of monomeric cations and aggregated species. In the cases of Cu/Al-FER, the estimated molar percentage ratio of monomeric to aggregated coppers species is 58/42. For the Cu/Ti-FER, there is a larger contribution of aggregated copper species and the ratio of monomeric to aggregated species is 7/93. In these calculations it was assumed that in the beginning of the H_2_-TPR run, copper in all species is present only as Cu^2+^, thus the presented values are only the rough estimation. However, it should be noted that the results of such an estimation are consistent with the results of UV-vis DRS analysis (Figure 4), which showed higher contribution of aggregated copper oxide species in the Cu/Ti-FER sample comparing to Cu/Al-FER. The reduction of copper species in Cu/Al-ITQ-6 and Cu/Ti-ITQ-6 also occurs in the similar temperature ranges (Figure 5). The estimation of the copper species contribution in Cu/Ti-ITQ-6 shows domination of aggraded species (about 90%) with a significantly lower content of monomeric Cu^2+^ cations (about 10%). A narrow and sharp low-temperature reduction maximum with only small shoulder may indicate high homogeneity of the deposited aggregated copper oxide species. A very surprising reduction profile was obtained for the Cu/Al-ITQ-6 sample. Deconvolution of the reduction profile for the low and high temperature reduction peaks and comparison of the surface areas integrated under these peaks shows that more hydrogen was consumed for the reduction of Cu^+^ to Cu^0^ at higher temperatures than for both the reduction of Cu^2+^ to Cu^0^ in aggregated species as well as monomeric Cu^2+^ cations to Cu^+^ at lower temperatures. This surprising effect is possibly related to the presence of monomeric Cu^+^ cations in the Cu/Al-ITQ-6 sample in the beginning of the H_2_-TPR run. Such thermal reduction of Cu^2+^ to Cu^+^ that occurred during the sample outgassing has been reported in scientific literature [28]. The thermal reduction in a flow of helium, used in the samples outgassing step, shows high lability in the reduction of monomeric Cu^2+^ cations to Cu^+^ in the Cu/Al-ITQ-6 sample. Another interesting observation is related to the tendency of copper species to aggregation in Ti-zeolites. Possibly this is related to the different interaction of copper species within the Ti- and Al-zeolite framework. In the case of Al-zeolites, Cu/Al-FER and Cu/Al-ITQ-6, copper is present mainly as monomeric Cu^2+^ or Cu^+^ cations in ion-exchange positions. Such Coulombic interactions between copper cations and negatively charger zeolite frameworks are relatively strong and therefore sintering of copper species under calcination conditions is limited. The nature of copper cation interactions with the Ti-zeolite framework is much more complex. Part of the ≡Si–O–Ti≡ bridges can be hydrolyzed under treatment with the aqueous solution of copper salt with the formation of weakly acidic ≡Si–OH and ≡Ti–OH, that can act as ion-exchange sites [32]. Moreover, copper can be attached to ≡Si–O–Ti≡ bridges as a surface complex [32]. In these cases, copper is attached into the zeolite framework by weaker forces and therefore aggregation of copper species under calcination conditions is much easier compared to Al-zeolites.

#### 3.1.5. NH_3_-TPD

**Figure 6:**
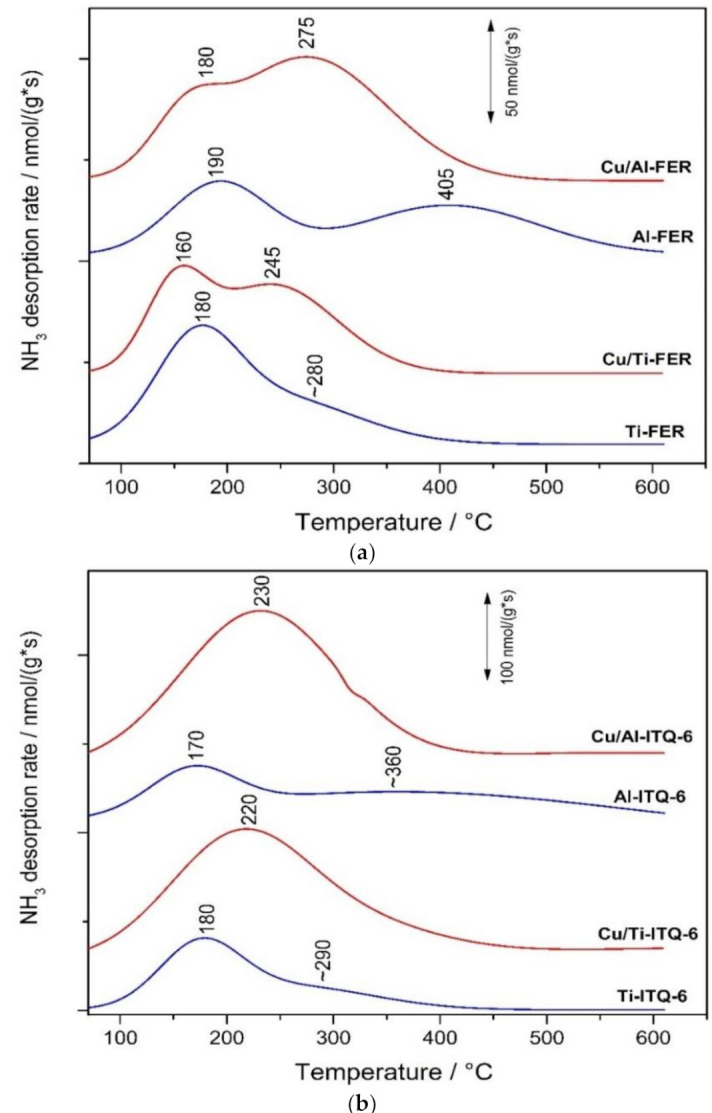
Profiles of NH_3_-TPD obtained for ferrierite (**a**) and ITQ-6 (**b**) zeolites.

Temperature-programed desorption of ammonia (NH_3_-TPD) was used for evaluation of the surface concentration and relative strength of acid sites. A complementary method based on FTIR analysis of the pyridine pre-adsorbed samples for determination of the Lewis and Brønsted acid sites contribution is not effective for ferrierites and ferrierite bases samples, because 10 MR channels in ferrierite are smaller than the kinetic diameter of pyridine, which is about 0.57 nm [33,34]. The ammonia desorption profiles of zeolites and their modifications with copper are presented in Figure 6, while the surface concentrations (C_a_) and densities (D_a_) of acid sites are compared in Table 1. It was assumed that one ammonia molecule is chemisorbed on one acid site and therefore the number of chemisorbed ammonia molecules directly indicates the number of acid sites located on the sample surface.

Ammonia desorption profile of Al-FER consists of two peaks centered at about 190 and 405 °C, indicating ammonia desorption from acid sites of weaker and stronger strength, respectively (Figure 6a). Substitution of aluminum by titanium in the Ti-FER framework resulted in disappearance of stronger acid sites. The position of the main low-temperature ammonia desorption peak, 180 °C, indicates the presence of slightly weaker acid sites comparing to sites present in Al-FER. In the case of Al-FER the role of acid sites could play –OH groups in ≡Si–O(H)–Al≡ bridges (Brønsted acid sites) and coordinative unsaturated Al^3+^ cations of the zeolite framework (Levis acid sites). The situation is more complex in the case of Ti-FER, where acid sites could be ≡Ti–OH groups, which according to Bordiga [35] can attach one or even to ammonia molecules. Moreover, ammonia molecules can be adsorbed on Ti^3+^ cations formed by e.g., charge relocation (O^2−^ Ti^4+^)→(O^−^ Ti^3+^). Significant modifications of ammonia desorption profiles were observed after copper deposition into Al-FER and Ti-FER (Figure 6a). Low-temperature peaks were slightly shifted to lower temperatures, indicating the formation of weaker acid sites. Moreover, additional peaks at about 245 and 275 °C appeared in the desorption profiles of Cu/Al-FER and Cu/Ti-FER, respectively. These peaks are possibly directly associated with deposited copper species and the difference in their positions is probably related to various types of copper species in the ferrierite samples—mainly monomeric copper cations in Cu/Al-FER and mainly aggregated copper oxide species in Cu/Ti-FER. It should be noted that the copper loading in Cu/Al-FER is three times lower compared to Cu/Ti-FER, but the concentration of acid sites in the former sample after copper deposition increased more significantly compared to the later one. This is not surprising taking into account the better surface accessibility of the copper species in the form of monomeric cations compared to aggregated copper oxide species. Ammonia desorption profiles of delaminated Al-ITQ-6 and Ti-ITQ-6 samples (Figure 6b) are similar to the profiles of Al-FER and Ti-FER (Figure 6a), respectively.

In the case of delaminated samples, the low-temperature peaks were slightly shifted to lower temperatures, indicating less acidic sites. Moreover, in the case of Al-ITQ-6, the high temperature maximum was broadened and shifted to lower temperatures, indicating increased heterogeneity of acid sites with respect to their strength. Introduction of copper into delaminated zeolites caused a significant increase in acid site concentration manifested by broad and intensive maxima centered at 230 and 220 °C for Cu/Al-ITQ-6 and Cu/Ti-ITQ-6, respectively. A comparison of ammonia desorption profiles of delaminated zeolites before and after copper deposition, shows that acid sites present in Cu/Al-ITQ-6 and Cu/Ti-ITQ-6 are associated mainly with deposited coppers species.

### 3.2. Catalytic Studies 

**Figure 7:**
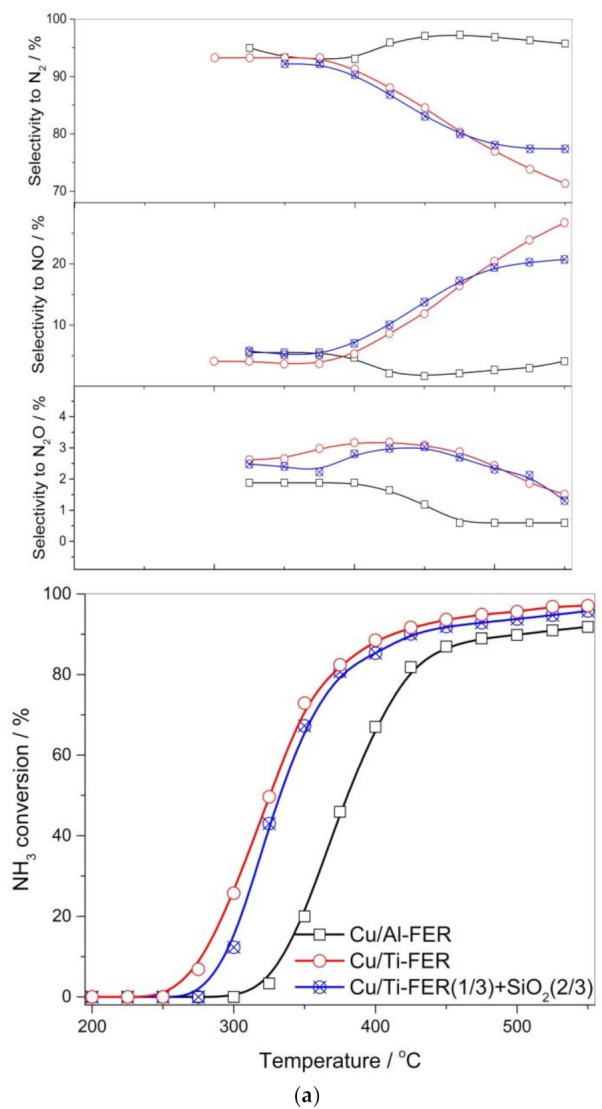

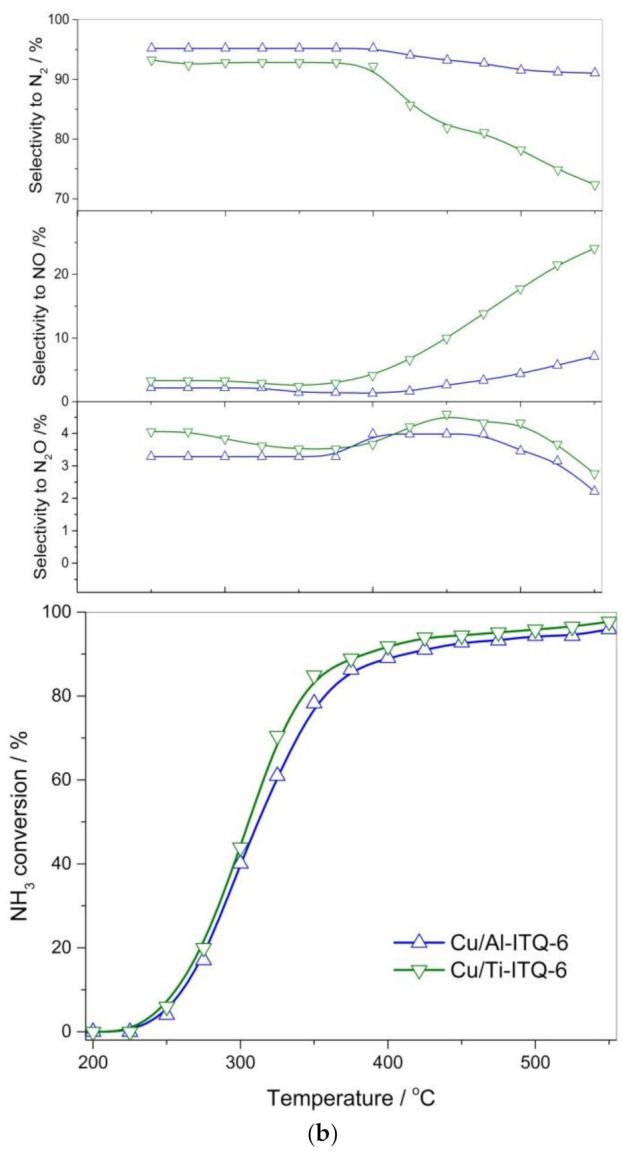
Results of NH_3_-SCO tests for ferrierite (**a**) and ITQ-6 (**b**) based catalysts.

Ferrierites and their delaminated forms doped with copper were studied as catalysts for the selective catalytic oxidation of ammonia (NH_3_-SCO). Dinitrogen is the desired reaction product, while N_2_O, NO and NO_2_ are the side reaction products. In the case of the studied catalysts, the only detected reaction products were N_2_, N_2_O and NO (Figure 7). NO_2_ was not detected in any catalytic run. Ammonia conversion over Cu/Al-FER started at a higher temperature than in the case of Cu/Ti-FER (Figure 7a). Dinitrogen was the main reaction product and selectivity to this product was above 90% in the whole studied temperature range for Cu/Al-FER, while in the cases of Cu/Ti-FER they decreased to about 70% at 550 °C. Such high selectivity to dinitrogen in the high temperature range, observed for Cu/Al-FER, is very promising. Such significant differences in the low-temperature activity of Cu/Ti-FER and Cu/Al-FER could be related to a three-times lower copper loading in the later catalyst (Table 1). In order to verify this hypothesis, the additional catalytic test for the sample containing 33 mg Cu/Ti-FER mixed with 67 mg of pure silica was done (Figure 7a). In this case, the content of copper in both samples, Cu/Ti-FER + SiO_2_ and Cu/Al-FER, was this same. As can be seen ammonia conversion over the Cu/Ti-FER + SiO_2_ sample still started at a significantly lower temperature than for Cu/Al-FER, indicating that copper loading was not a crucial parameter determining catalytic performance of the Cu/Ti-FER and Cu/Al-FER samples. Thus, it seems that form and aggregation of deposited copper species determine catalytic activity of the samples in the NH_3_-SCO process. As it was shown by UV-Vis DRS (Figure 7b) and H_2_-TPR (Figure 5) studies that in Cu/Al-FER dominated monomeric copper species, while in Cu/Ti-FER aggregated copper oxide species. Thus, it is postulated that aggregated copper species are more active in ammonia oxidation compared to monomeric copper cations. Similar conclusions have been already reported in scientific literature [8,18]. Another very significant difference is related to the high temperature selectivity to dinitrogen, which is significantly lower for Cu/Ti-FER than for Cu/Al-FER (Figure 7a). The main side product of the high temperature ammonia oxidation conducted in the presence of Cu/Ti-FER is NO. Such significant differences in selectivities of ammonia oxidation products could be explained assuming the mechanism of internal selective catalytic reduction (i-SCR), including direct oxidation part of ammonia to NO in the first step and reduction of NO with residual ammonia to dinitrogen and water vapor (similarly to classical NH_3_-SCR). Thus, effective catalysts for the NH_3_-SCO process should contain components active in ammonia to NO oxidation as well as in reduction of NO with ammonia to N_2_ and H_2_O. Too high an activity in the first step may result in complete ammonia oxidation to NO and in such a case in the second step, resulting in dinitrogen being impossible. Aggregated copper oxide species were reported to be catalytically active in ammonia to NO oxidation [18] and therefore activity of Cu/Ti-FER at lower temperatures is not surprising. Highly dispersed coppers species are less active in the low-temperature ammonia oxidation [18] but have been reported as effective catalysts of the selective catalytic reduction of NO with ammonia (NH_3_-SCR) [17], which may explain the activity of Cu/Ti-FER at higher temperatures, but also very high selectivity to dinitrogen. It should be also noted that the NH_3_ conversion did not reached 100%, even at high temperatures, indicating that ammonia is available for the second step of i-SCR mechanism. Delaminated zeolites modified with copper, Cu/Al-ITQ-6 and Cu/Ti-ITQ-6, presented a significantly improved catalytic activity comparing to ferrierites doped with copper. Ammonia conversion started at about 200 °C and the level of 90% was obtained at about 400 °C for Cu/Ti-ITQ-6 and 425 °C for Cu/Al-ITQ-6 (Figure 7b). Thus, the catalysts based on delaminated zeolites presented better catalytic activity than ferrierites modified with copper. Similar to the ferrierite catalysts and also for the ITQ-6 series, better catalytic activity, manifested by conversion of ammonia at lower temperature, was observed for catalysts containing titanium incorporated into the zeolite framework (Figure 7b). However, this difference is less significant compared to the ferrierite series (Figure 7a). Cu/Ti-ITQ-6, as it was determined by H_2_-TPR (Figure 5), contains mainly aggregated copper oxide species with a significant contribution of monomeric copper cations. Thus, assuming i-SCR mechanism, copper oxide aggregates are possibly active in NH_3_ to NO oxidation, while highly dispersed copper species are catalytically active in NO reduction with residual ammonia to N_2_ and H_2_O. At a temperature above 400 °C, the selectivity to dinitrogen decreased possibly due to a higher contribution of aggregated copper species. The ammonia oxidation over Cu/Al-ITQ-6, containing copper mainly in the form of monomeric copper cations, started at higher temperatures but also the selectivity to dinitrogen was above 90% in the studied temperature range. Thus, these observations support the concept of the i-SCR mechanism also for Cu/Al-ITQ-6 and Cu/Ti-ITQ-6. The increased activity of these catalysts compared to Cu/Al-FER and Cu/Ti-FER is possibly related to better accessibility of copper species. Analysis of the ammonia desorption profiles of Cu/Al-ITQ-6 and Cu/Ti-ITQ-6 (Figure 6b) shows that the majority of acid sites was generated by copper deposited into Al-ITQ-6 and Ti-ITQ-6. If we assume that interaction between copper and ammonia is based on accommodation of a free electron pair of ammonia into uncopied d-orbitals of copper (NH_3_→Cu^n+^), the number of surface-exposed copper cations could be roughly estimated from NH_3_-TPD profiles. The majority of the ammonia oxidation pathways include chemisorption and activation of ammonia molecules as one of the reaction steps. The concentration of ammonia chemisorbed on Cu/Al-ITQ-6 and Cu/Ti-ITQ-6 is much higher than for other samples (Figure 6b, Table 1), indicating higher accessibility of the surface copper cations for catalytic ammonia conversion. This is not surprising because in delaminated zeolites, copper aggregates can be located not only on the external surface of zeolite grains or crystallites, but also in the interlayer space of ITQ-6 zeolites. The Cu/Ti-ITQ-6 sample, as it was shown in Table 1, is characterized by a significantly higher BET surface area and mesopore volume than other copper-containing catalysts. As was postulated, the increased values of these textural parameters are associated with the presence of amorphous silica deposited on the zeolite crystallites during the swelling process. Deposition of copper by the ion-exchange method resulted possibly in the preferential location of copper cations in ion-exchange positions; however, deposition of copper on the amorphous silica surface cannot also be excluded. Our previous studies for MCM-41 modified with copper showed its relatively high catalytic activity in the NH_3_-SCO process [31]. Thus, the contribution of copper species deposited on amorphous silica in the overall catalytic performance of Cu/Ti-ITQ-6 cannot be excluded.

## 4. Conclusions 

Copper modified ferrierites and their delaminated forms presented very promising catalytic properties in the selective catalytic oxidation of ammonia to dinitrogen. It was shown that both forms of deposited copper species as well as porous structure of zeolites play a very important role in the catalytic performance of the studied samples. Deposition of copper into Al-FER and Al-ITQ-6 resulted in highly dispersed species, mainly monomeric copper cations. On the other side, introduction of copper into Ti-FER and Ti-ITQ-6 resulted mainly in aggregated copper oxide species. This effect was explained by the less-effective stabilisation of copper species deposited into Ti-zeolites, which under calcination conditions were sintered with the formation of more aggregated species. In the case of Al-zeolites, copper cations located in ion-exchange sites are more effectively stabilised and less susceptible to aggregation. In the case of Cu/Al-FER, the copper loading is about three times lower compared to Cu/Ti-FER (Table 1), which also resulted in the lower contribution of aggregated copper oxide species in these samples. For the delaminated Cu/Al-ITQ-6 and Cu/Ti-ITQ-6 samples, the contribution of dispersed copper species is higher than in the analogues microporous ferrierite samples, Cu/Al-FER and Cu/Ti-FER, which is possibly related to the accessibility of the interlayer space for copper deposition. In the case of ferrierites, copper deposition inside pores are limited due to the similar size of octahedral aqua copper complexes and 10 MR channels in zeolite. Therefore, internal diffusion of such complexes decreased the efficiency of copper deposition in the form of monomeric copper cations inside pores. 

It is postulated that copper oxide aggregates are catalytically active in direct ammonia oxidation to NO, while dispersed copper species are catalytically active in NO reduction with ammonia to dinitrogen. Assuming that the ammonia oxidation process over studied catalysts could be described by internal selective catalytic reduction (i-SCR) mechanism, the role of different types of copper species can be explained. In the case of Ti-zeolites, copper is present mainly in the form of aggregated species, catalytically active in the low-temperature ammonia oxidation. An increase in the selectivity to NO and a decrease in the selectivity to N_2_, observed at higher temperatures, is a result of nearly complete direct ammonia conversion to NO and therefore ammonia shortage for the second step of i-SCR mechanism—selective reduction of NO with residual ammonia to dinitrogen. On the other hand, there is a significant contribution of copper in the form of monomeric cations in Al-zeolites. Due to the lower content of copper oxide aggregates in these catalysts, ammonia conversion is observed at higher temperatures, but selectivity to dinitrogen is very high in the whole studied temperature range. Thus, in the case of Cu/Al-zeolites, ammonia was not completely oxidised to NO, and therefore residual ammonia may reduce NO to dinitrogen in the presence of highly dispersed copper species, catalytically active in this process.

## Figures and Tables

**Table 1:** Textural parameters, surface acidity, copper content, as well as molar Si/Al and Si/Ti ratios in zeolitic samples.

Sample	S_BET_ (m^2^/g)	V_micro_ (cm^3^/g)	V_meso_ (cm^3^/g)	C_a_ (µmol/g)	D_a_ (µmol/m^2^)	Cu (wt%)	Si/Al (mol/mol)	Si/Ti (mol/mol)
**Ti-FER**	368	0.14	0.09	49	0.13	-		60
**Al-FER**	377	0.13	0.10	49	0.13		64	
**Ti-ITQ-6**	615	0.02	1.38	70	0.11	-		80
**Al-ITQ-6**	372	0.02	1.12	37	0.10		111	
**Cu/Ti-FER**	330	0.12	0.09	54	0.16	2.7		68
**Cu/Al-FER**	359	0.12	0.11	79	0.22	0.9	74	
**Cu/Ti-ITQ-6**	523	0.02	0.78	141	0.27	2.8		85
**Cu/Al-ITQ-6**	281	0.02	0.47	162	0.58	2.7	124	
